# Skeletal Muscle Mass, Strength, and Functional Status in Critically Ill Older Adults Aged 60–75 and over 80 Years Old: An Exploratory Study

**DOI:** 10.3390/healthcare14050585

**Published:** 2026-02-26

**Authors:** Ruud Teppa-Zyl, L. Felipe Damiani, Rodrigo Muñoz-Cofre, Carolina Carmona-Valenzuela, Gabriel Nasri Marzuca-Nassr

**Affiliations:** 1 Hospital Dr. Hernán Henríquez Aravena, Manuel Mont 115, Temuco 4780000, Chile; 2 Escuela de Ciencias de la Salud, Departamento de Kinesiología, Facultad de Medicina, Pontificia Universidad Católica de Chile, Santiago 7820436, Chile; 3 Unidad de Enfermedades Respiratorias, Departamento de Medicina Interna, Facultad de Medicina, Universidad de La Frontera, Claro Solar 115, Temuco 4780000, Chile; 4 Departamento de Ciencias de la Rehabilitación, Facultad de Medicina, Universidad de La Frontera, Claro Solar 115, Temuco 4780000, Chile

**Keywords:** elderly, muscle weakness, critical care, muscular atrophy, functional status

## Abstract

**Background:** Older adults are highly heterogeneous, spanning from robust to frail. Older adults aged ≥80 years (older 80+ group) admitted to an intensive care unit (ICU) present with greater severity and face higher risks of functional decline and mortality than younger ICU patients. **Objective**: This study compared the trajectory of skeletal muscle mass, strength, and functional status between ICU patients aged 60–75 years and those in the older 80+ group. **Methods:** A non-concurrent prospective observational study was conducted in older adults who were admitted to an ICU. Skeletal muscle mass, muscle strength and functional status from ICU admission to discharge were assessed. **Results:** Regarding muscle mass, no differences were observed at ICU admission in patients aged 60–75 years and those in the older 80+ group. However, both groups experienced a decrease in quadriceps muscle thickness (Time Effect: vastus intermedius (VI): *p* < 0.001; rectus femoris (RF): *p* < 0.001; and total quadriceps (TQ): *p* < 0.001) during their ICU stay. No differences were observed at awakening in handgrip strength (HS), Medical Research Council Sum Score (MRC-SS) and Functional Status Score for the Intensive Care Unit (FSS-ICU) between groups. However, HS, MRC-SS, and the FSS-ICU scale all increased from awakening to ICU discharge (Time Effect HS: *p* = 0.002; MRC-SS: *p* < 0.001; and FSS-ICU: *p* < 0.001). **Conclusions:** Both older adult groups admitted to the ICU experience a decline in skeletal muscle mass, muscle strength, and functional status during their ICU stay, with a tendency for the decline to be greater in the older 80+ group. In addition, critically ill older groups recover muscle strength and functional status from awakening to ICU discharge, with a tendency for the recovery to be smaller in the older 80+ group. These conclusions are based on trends observed in an exploratory study and require confirmation in larger trials.

## 1. Introduction

The aging of the population is a rapidly accelerating global phenomenon, particularly in developing countries [1]. By 2050, it is estimated that one in six people worldwide will be over 65 years old, and the number of older adults aged ≥80 years (older 80+ group) will increase threefold. The growing presence of critically ill older adults has progressively contributed to changing the profile of patients admitted to intensive care units (ICUs) [2], where it is estimated that approximately 15% of ICU patients are in the older 80+ group, and 1% are 90 years or older [3].

Older adults represent a highly heterogeneous group, where individuals with high functional status reserves coexist alongside others who are frail. Several studies indicate that adults aged 80 and older are admitted to ICUs with greater severity and face a higher risk of functional status decline and mortality compared with younger ICU patients [4,5]. This phenomenon is often multifactorial, associated with an underlying inflammatory process combined with immobilization, disuse, hyperglycemic states, microcirculatory disorders, and bioenergetic impairment, among other factors [6,7,8]. Older adults in ICUs experience significant skeletal muscle mass loss early characterized by an increased catabolic state and depressed anabolism; mechanical ventilation further accelerates protein degradation, causing diaphragmatic atrophy and widespread muscle dysfunction [9]. This condition is accompanied by a decline in muscle strength, physical performance, and functionality, ultimately reducing quality of life [10].

Within the older adult population, age gaps exceeding 20 years are common (i.e., “younger-old” adults aged 60–80 years versus “oldest-old” adults aged >80 years). Such gaps magnify aging-related heterogeneity and may translate into clinically meaningful divergence in baseline characteristics and prognosis [11]. In this context, individuals in the older 80+ group constitute a particularly vulnerable subgroup, with lower physiological reserve and a higher burden of frailty and multimorbidity [12]. Regarding body composition, skeletal muscle mass declines by approximately one third after age 50 and decreases by an additional ~15% between ages 70 and 80, together with a higher prevalence of sarcopenia among those in the older 80+ group (up to ~50%) [13]. Moreover, advancing age intensifies inflammaging—a chronic, low-grade inflammatory state—which may exacerbate muscle loss and delay recovery following the catabolic stress associated with critical illness, especially among individuals in the older 80+ group [14]. In the ICU setting, these factors may amplify baseline differences across older adult subgroups and shape distinct trajectories of deterioration and recovery, an area that remains insufficiently characterized.

Currently, several studies evaluating older adults in the ICU have primarily focused on physical function after ICU discharge [15,16], resulting in limited understanding of their functional trajectory during the ICU stay. Moreover, most of these studies have grouped all older patients into a single category, without examining potential differences across age subgroups.

Thus, the objective of the present pilot study is to characterize the progression of skeletal muscle mass, muscle strength, and functional status in ICU patients in the older 80+ group, and to compare these outcomes with those of ICU patients aged 60 to 75 years who require mechanical ventilation. This approach seeks to identify both baseline differences and changes occurring during the ICU stay, with the aim of detecting preventable complications and informing the development of future studies.

## 2. Methodology

### 2.1. Aim

This study’s aim was compare the trajectory of skeletal muscle mass, strength, and functional status between ICU patients aged 60–75 years and those in the older 80+ group.

### 2.2. Study Design

A non-concurrent prospective observational study was conducted on critically ill older adults admitted to the Adult Critical Care Unit (medical and surgical) at Dr. Hernán Henríquez Aravena Hospital in Temuco, Chile. The study received approval from the Scientific Ethics Committee of the Araucanía Sur Health Service (file number 249). All participants provided retrospective informed consent once they were awake and clinically stable.

### 2.3. Participants

Adult patients aged 60 years or older, requiring at least 72 h of mechanical ventilation, were included. We defined the age strata a priori to compare two non-overlapping age groups with clearer biological and clinical distinctions: patients aged 60–75 (younger-old) and the older 80+ group (very old). Participants aged 76–79 years were not included to maintain a minimum 5-year separation between strata and to avoid an intermediate age range that could reduce between-group contrast and increase misclassification in an exploratory study.

Patients with neurological diagnoses, severe dependency at admission (determined at the initial assessment based on clinical and functional information and patient clinical history), pre-existing physical or cognitive disorders (as per medical records), or an extremely critical condition—defined as multiorgan dysfunction requiring maximal life support (mechanical ventilation, vasopressors and/or inotropes) or extracorporeal membrane oxygenation (ECMO)—were excluded.

All patients received standard physical therapy consisting of passive range-of-motion mobilization of the upper and lower limbs (flexion and extension) while the patient was sedated in the ICU. Upon awakening, care continued with the unit’s standard mobilization protocol, progressing through postural changes and motor milestones.

A total of thirty-five participants aged 60 years or older were initially eligible during the study period. Eight patients were excluded due to requiring less than 72 h of mechanical ventilation whereas ten were excluded because of a neurological deficit, motor disorder, or death. A total of 17 patients were finally included in the analysis, with eight participants in the group aged 60–75 years (3 men and 5 women; age: 68 ± 6 years; body mass index (BMI): 30.04 ± 5.14 kg/m^2^) and nine participants in the older 80+ group (5 men and 4 women; age: 84 ± 4 years; BMI: 29.42 ± 2.70 kg/m^2^) (Supplementary Figure S1).

This was an exploratory study with consecutive enrollment of all eligible patients admitted between June 2020 and June 2021. Given the exploratory nature and feasibility constraints in this ICU population, no formal power-based sample size was used to determine enrollment. Effect sizes with confidence intervals are reported to quantify the magnitude of changes and to inform sample size planning for future confirmatory studies.

### 2.4. Procedures

Once patients were admitted to the ICU and enrolled in the study, demographic and clinical variables were registered from clinical records. Also, different muscle characteristics including the evolution of skeletal muscle mass (quadriceps femoris thickness) were evaluated at ICU admission, awakening, and ICU discharge. Additionally, handgrip strength, peripheral muscle strength and functional status were evaluated when patients were able to collaborate during awakening and at the ICU discharge. ICU admission corresponded to the evaluation performed within the first 24 h of admission to the unit. Awakening assessment was conducted when the patient reached a score of ≥3 out of 5 on the “Standardized Five Questions” (S5Q). This tool, through five simple questions—”open/close your eyes,” “look at me,” “open your mouth and stick out your tongue,” “nod your head,” and “raise your eyebrows when I count to 5”—assesses the level of cooperation and is widely used in ICU settings [17,18]. A minimum response of 3 out of 5 questions identified the patient as “able to cooperate”. This assessment was conducted daily. Finally, all assessments at ICU discharge were performed 12 h before ICU discharge. Supplementary Figure S2 details the different assessments conducted during the ICU stay.

### 2.5. Skeletal Muscle Mass

For muscle mass assessment, quadriceps muscle thickness (vastus intermedius and rectus femoris) was measured using muscle ultrasonography. Images were obtained with a portable ultrasound device, the NextGen LOGIQ e Ultrasound from General Electric Company©, GE Healthcare, Chicago, IL, USA. A 9 MHz linear transducer was used for this measurement. The device settings included gain, depth, and mode adjustments. The same researcher performed the quadriceps muscle thickness evaluation at admission, awakening, and discharge from the unit. For quadriceps thickness measurement, the reference point used was the midpoint between the anterior superior iliac spine and the superior pole of the patella. The transducer was positioned perpendicular to the long axis of the thigh. Participants were evaluated in a supine position with their arms and legs extended, in a neutral rotation, and with muscles completely relaxed. A generous amount of contact gel was applied to minimize the pressure required by the transducer on the skin [19]. At the reference point, the image was frozen, and measurements were taken as follows: (1) from the femoral cortex to the fascia separating the vastus intermedius (VI) from the rectus femoris (RF), obtaining the VI muscle thickness; (2) from the fascia separating the VI from the RF to the beginning of the superior fascia of the RF, obtaining the RF muscle thickness; (3) from the femoral cortex to the superior fascia of the RF to obtain the total quadriceps thickness (TQ), according to previous protocols [18]. The measurements were recorded in centimeters (cm). For each muscle, three consecutive measurements were taken, and the average value was recorded. To document the robustness of ultrasound measurements performed by a single examiner, intra-rater reliability and measurement error were assessed using repeated measurements on stored ultrasound images. The same examiner re-measured muscle thickness on four stored images on two separate occasions (T1 and T2), blinded to the initial results, using the mean value according to the study protocol. Intra-rater reliability was excellent (ICC, two-way mixed-effects model, absolute agreement = 0.982). Measurement error was low, with a standard error of measurement (SEM) of 0.026 and a minimal detectable change at the 95% confidence level (MDC95) of 0.073 (same units as muscle thickness).

### 2.6. Handgrip Strength

The assessment of handgrip strength (HS) was conducted using a JAMAR® Plus+ Digital Hand Dynamometer (Performance Health, Downers Grove, IL, USA). The protocol proposed by the American Society of Hand Therapists, as described in previous studies, was followed [20]. Both dominant and non-dominant handgrip strength was assessed. The same researcher conducted the evaluations upon awakening and at ICU discharge. During the assessment, the participant was positioned in a long sitting posture with shoulders adducted and without rotation, the elbow flexed at 90° at the side of the body, and the forearm and wrist in a neutral position. The evaluated arm was supported on a pillow. The researcher held the dynamometer in a vertical position, and the participant exerted their maximum grip strength for 3 s. A 30 s rest period was allowed between each trial. Handgrip was measured three times for each hand alternately, and the highest recorded value was used for analysis [18]. The evaluation was conducted when the patient was alert and cooperative, with an S5Q score of ≥3 points.

### 2.7. Peripheral Muscle Strength

The Medical Research Council Sum Score (MRC-SS) evaluates the global peripheral muscle strength of six major muscle groups during functional movements (shoulder abduction, elbow flexion, wrist extension, hip flexion, knee extension, and ankle dorsiflexion) in each hemi-body, classifying them between 0 (no visible contraction) and 5 (normal strength in the full range of motion) [21]. Each participant was assigned a score ranging from 0 (total paralysis) to 60 (normal strength). Evaluations were performed from right to left and from proximal to distal, following the same order. Up to three attempts were considered optimal for each muscle group, with a rest period of at least 30 s between attempts [22].

### 2.8. Functional Status

The assessment of functional status was conducted using the Chilean version of the Functional Status Score for the Intensive Care Unit (FSS-ICU) [23]. The assessment was conducted by the same researcher at awakening and at ICU discharge. The FSS-ICU measures the level of physical assistance required for five functional activities in the ICU setting: rolling, transitioning from supine to sitting, sitting at the edge of the bed, transferring from sitting at the edge of the bed to standing, and walking. Walking was assessed only if the participant was able to complete a 30 m walk. Each activity is scored from 0 points (inability to perform) to 7 points (complete independence). The total score is the sum of the scores for each completed activity, with a higher score indicating greater functional mobility [23].

### 2.9. Statistics

Data were tabulated in Microsoft Excel^®^ and analyzed using IBM SPSS Statistics 30.0^®^. Figures were created using GraphPad Prism^®^ 10.3.1 software (San Diego, CA, USA). Results are presented as mean ± standard deviation for continuous variables and as frequency and percentage for categorical variables. Main results and estimates are reported with 95% confidence intervals (95% CIs). Normality was assessed using the Shapiro–Wilk test for each variable by group and measurement time point (Supplementary Table S3). Assumptions for repeated-measures ANOVA were evaluated prior to inferential testing; sphericity was examined using Mauchly’s test, and when the assumption of sphericity was violated (*p* < 0.05), the Greenhouse–Geisser correction was applied to adjust the degrees of freedom. The independent-sample *t* test was then used to compare group means (e.g., baseline characteristics), and the repeated-measures ANOVA was used to assess the effect of time and group and the interaction between patients aged 60 to 75 years and those in the older 80+ group. If there was an interaction, the dependent *t* test was used. The differences or delta changes in outcome variables between ICU admission and ICU discharge and between awakening and ICU discharge were also calculated as percentages and compared using the independent-sample *t* test. A *p* value < 0.05 was considered statistically difference.

## 3. Results

### 3.1. Participants’ Characteristics

A total of 35 participants were initially evaluated: 19 aged 60–75 years, of whom 11 were excluded, and 16 in the older 80+ group, of whom 7 were excluded (Supplementary Figure S1). Table 1 shows the baseline characteristics of 17 patients included in the final analysis; significant differences were observed only for age. Deaths were attributed to disease severity. All participants admitted to the study required mechanical ventilation and sedation/analgesia from the time of admission to the ICU, and only one participant aged 60–75 years required neuromuscular blockade. Regarding pharmacological management, all participants received sedation (fentanyl, midazolam, and propofol), vasoactive agents, and routine medications, including anticoagulants, analgesics, and gastrointestinal prophylaxis. Antibiotics were administered to 82% of patients (7 aged 60–75 years and 7 in the older 80+ group), whereas 58% received corticosteroids (4 aged 60–75 years and 6 in the older 80+ group) and 53% received bronchodilators. Neuromuscular blockade was used in only one participant aged 60–75 years.

### 3.2. Skeletal Muscle Mass

**Figure 1:**
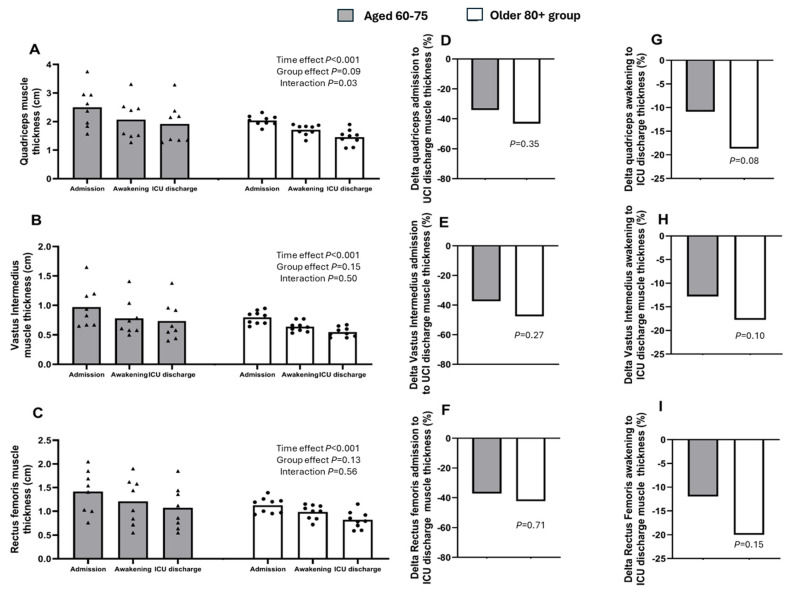
(**A**–**C**) Quadriceps, vastus intermedius, and rectus femoris muscle thickness measured from ICU admission to ICU discharge in both groups, respectively. (**D**–**F**) Percentage change in quadriceps, vastus intermedius, and rectus femoris muscle thickness from ICU admission to ICU discharge in both groups, respectively. (**G**–**I**) Percentage change in quadriceps, vastus intermedius, and rectus femoris muscle thickness from awakening to ICU discharge in both groups, respectively. Aged 60–75 years, n = 8; older 80+ group, n = 9.

During their ICU stay (14 ± 10 days for participants aged 60–75 years and 11 ± 4 days for the older 80+ group), both groups of older adults experienced a decrease in quadriceps muscle thickness from admission to discharge (2.50 ± 0.71 vs. 1.92 ± 0.71 cm in those aged 60–75 years and 2.03 ± 0.17 vs. 1.45 ± 0.26 cm in the older 80+ group, respectively), with no differences between the groups (Figure 1A).

The percentage loss in quadriceps muscle thickness from ICU admission to ICU discharge in the older 80+ group, compared with that in those aged 60–75 years, was as follows: vastus intermedius: −47.56 ± 19.83% vs. −37.11 ± 18.22%, *p* = 0.27 [95% CI, −5.40–19.22]; rectus femoris: −42.48 ± 33.57% vs. −37.30 ± 21.33%, *p* = 0.71 [95% CI, −17.73–26.61]; and total quadriceps: −43.16 ± 19.91% vs. −34.03 ± 19.34%, *p* = 0.35 [95% CI, −29.47–11.26] (Figure 1D–F). Similarly, the percentage loss observed from awakening to ICU discharge was: vastus intermedius: −12.80 ± 13.92% vs. −17.73 ± 9.75%, *p* = 0.10 [95% CI, −1.58–14.47]; rectus femoris: −11.94 ± 7.13% vs. −20.89 ± 14.16%, *p* = 0.15 [95% CI, −3.46–20.26]; and total quadriceps: −8.9 ± 5.45% vs. −18.7 ± 10.32%, *p* = 0.08 [95% CI, −1.15–16.80] (Figure 1G–I).

### 3.3. Handgrip Strength

**Figure 2:**
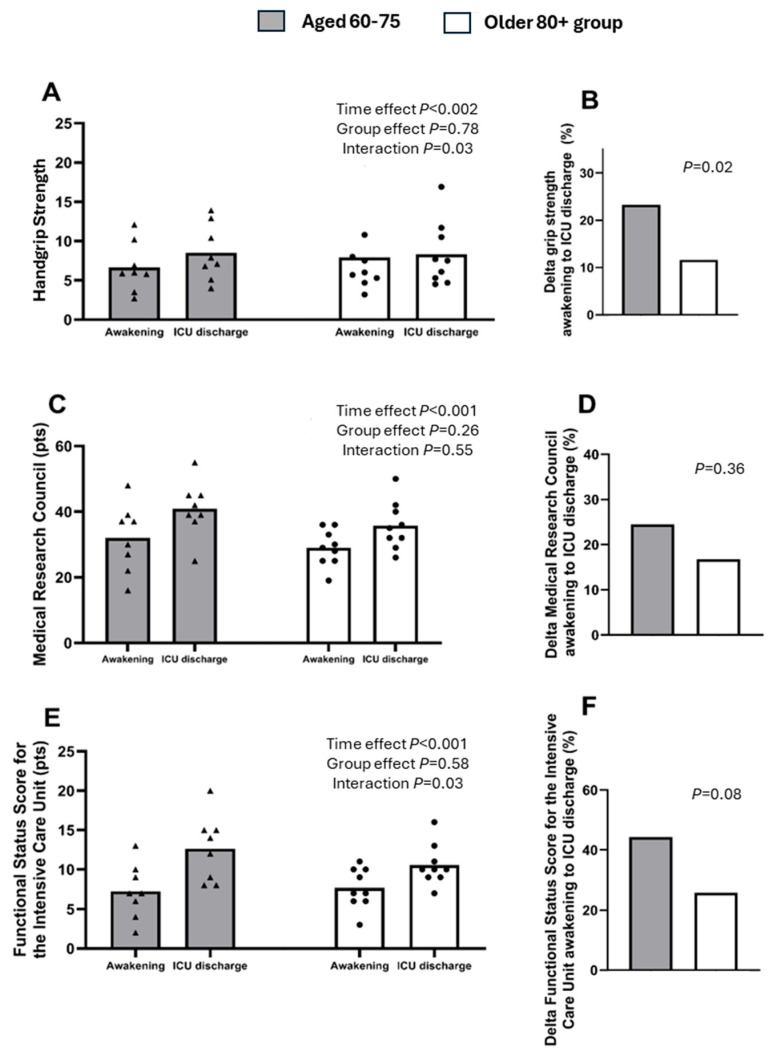
(**A**) Handgrip strength (HS) measured at awakening and at ICU discharge in both groups. (**B**) Delta increase in HS from awakening to ICU discharge in both groups. (**C**) Medical Research Council Sum Score (MRC-SS) measured at awakening and at ICU discharge in both groups. (**D**) Delta increase in MRC-SS from awakening to ICU discharge in both groups. (**E**) Functional Status Score for the ICU (FSS-ICU) measured at awakening and at ICU discharge in both groups. (**F**) Delta increase in FSS-ICU from awakening to ICU discharge in both groups. Aged 60–75 years, n = 8; older 80+ group, n = 9.

At the time of awakening in the ICU, older adults aged 60–75 years had a handgrip strength of 6.63 ± 3.15 kg, compared with those in the older 80+ group, which had an HS of 7.91 ± 5.03 kg (*p* = 0.54 [95% CI, −3.13–5.68]). From awakening to ICU discharge, both groups experienced an increase in handgrip strength (Time Effect: *p* = 0.002). At ICU discharge, older adults aged 60–75 years had an HS of 8.51 ± 3.56 kg, compared to 8.32 ± 4.07 kg in the older 80+ group (*p* = 0.91 [95% CI, −4.17–3.79]). The percentage increase in handgrip strength from awakening to discharge was significantly lower in the older 80+ group compared to the group aged 60–75 years (7.91 ± 14.50% vs. 23.18 ± 9.02%; *p* = 0.02 [95% CI, −27.96–−2.57], Figure 2B).

### 3.4. Peripheral Muscle Strength

At the time of awakening in the ICU, older adults aged 60–75 years had a score of 32 ± 10.28 points on the MRC-SS scale, compared to the older 80+ group, which scored 29 ± 5.56 points (*p* = 0.45 [95% CI, −5.40–11.40]). During the ICU stay, both groups showed an increase in peripheral muscle strength on the MRC-SS scale (Time Effect: *p* < 0.001), with no significant differences between groups (*p* = 0.26 [95% CI, −3.09–13.28]).

The percentage increase in peripheral muscle strength, as measured by the MRC-SS scale, was 24.35 ± 17.0% in participants aged 60–75 years, compared with 16.00 ± 19.33% in the older 80+ group (*p* = 0.36 [95% CI, −27.29–10.59], Figure 2D).

### 3.5. Functional Status

At the time of awakening in the ICU, participants aged 60–75 years had a Functional Status Score of 7.25 ± 3.45 points on the FSS-ICU scale, compared with the older 80+ group, who scored 7.67 ± 2.55 points (*p* = 0.77 [95% CI, −2.69–3.53]).

During the ICU stay, both groups showed an increase in functional status on the FSS-ICU scale (Time Effect: *p* < 0.001), with no significant differences between the groups (*p* = 0.58; Figure 2E). At ICU discharge, participants aged 60–75 years had a Functional Status Score of 12.63 ± 4.20 points on the FSS-ICU scale, compared with 10.56 ± 2.60 points in the older 80+ group (*p* = 0.23 [95% CI, −5.63–1.49]). The percentage of functional status improvement in participants aged 60 –75 years was 44.44 ± 15.82%, compared to 26.00 ± 23.33% in the older 80+ group (*p* = 0.08 [95% CI, −39.34–2.46], Figure 2F).

## 4. Discussion

The aim of this study was to characterize and compare skeletal muscle mass, muscle strength, and functional status in older adults aged 60 to 75 years and those in the older 80+ group who were admitted to the ICU.

In this pilot study, we observed that critically ill patients in the older 80+ group had lower quadriceps muscle mass upon admission compared with critically ill patients aged 60–75 years (2.04 ± 0.14 cm vs. 2.44 ± 0.77 cm, respectively), although this difference did not reach statistical significance, likely due to the small sample size. Additionally, we confirmed a sustained decrease in the thickness of the vastus intermedius, rectus femoris, and total quadriceps in both groups throughout the ICU stay, with a tendency for the decline to be greater in the older 80+ group (Supplementary Figure S3). Similarly, strength, assessed by handgrip strength and the MRC-SS scale, along with functional status, assessed by the FSS-ICU scale, increased from awakening to ICU discharge, with a tendency to be smaller in the older 80+ group.

To our knowledge, this is the first attempt to characterize and compare the progression of skeletal muscle mass, muscle strength, and functional status in ICU patients aged 60 to 75 years versus those in the older 80+ group who require mechanical ventilation. In this study, quadriceps femoris muscle thickness exhibited a daily loss of ~2% in participants aged 60–75 years, compared to ~4% in the older 80+ group (Supplementary Table S1).

It has been reported that seven days of muscle disuse in healthy young subjects leads to skeletal muscle mass loss of 0.6% per day in the lower limbs [24]. Similarly, a 23% smaller thigh cross-sectional area (CSA) has been observed in healthy community-dwelling women over 80 years old compared to young women [25].

Other authors [6,18,26] have reported values of quadriceps muscle thickness loss between 16% and 18% in critically ill patients during their ICU stay, with an average reduction of approximately 1.5% to 2% per day, which would continue progressively throughout the ICU stay. The authors also noted that patients aged ≥60 years or those on mechanical ventilation for more than 10 days experience greater skeletal muscle mass loss [18].

In this regard, 30% reductions in the thickness of the rectus femoris and vastus intermedius, as well as in the CSA of the rectus femoris, have been reported during the first 10 days in an ICU [27]. These values are closer to those reported in our study, where we found skeletal muscle mass loss of 37% in participants aged 60–75 years for both the rectus femoris and vastus intermedius and 34% for the total quadriceps. For the older 80+ group, the loss values obtained were 42% and 47% for the rectus femoris and vastus intermedius, respectively. We believe that the more pronounced difference observed across studies may be related to the characteristics of the participants in our study, who had an older age range compared to those in previous studies by Andrade-Junior et al. [26], Fazzini et al. [6], Silva-Gutiérrez et al. [18], and Parry et al. [27]. These studies report younger populations, some starting from 15 years of age, with average ages closer to those of participants aged 60–75 years in our study. In particular, the systematic review by Fazzini et al. [6], which included a total of 52 studies, showed that many of them excluded individuals in the older 80+ group, limiting the availability of reference data to compare our results in this age group.

It is expected that baseline differences and divergent trajectories of muscle loss will be observed between the studied groups. Population-based evidence indicates a progressive decline in lean mass [13] and an increasingly pronounced deterioration in strength and power at advanced ages, supporting meaningful differences between these two older cohorts [28]. In addition, mechanisms inherent to advanced aging, such as anabolic resistance and inflammaging [14], may amplify the effects of the catabolic stress and immobilization characteristic of ICUs, thereby promoting greater muscle loss and/or slower recovery in the older 80+ group.

Furthermore, the higher burden of multimorbidity and geriatric syndromes among adults in the older 80+ group is associated with alterations in metabolic substrate utilization and in the function of key tissues (skeletal muscle, adipose tissue, and liver tissues), with dysregulation of glucose and lipid metabolism; this may translate into reduced metabolic “flexibility” and a more constrained response to acute stressors [29].

When analyzing our sample of participants, approximately 50% of participants aged 60–75 years and around 55% of the older 80+ group were admitted with a diagnosis of septic shock. Sepsis is characterized by a decrease in skeletal muscle mass, a reduction in muscle fiber size, and a decrease in muscle strength, which occurs early, starting on day 2, compared with disuse muscle atrophy, which is detectable on day 7 [30]. This factor may have influenced the skeletal muscle mass loss observed in both groups.

In addition, in the older 80+ group, two participants were admitted due to burn-related injuries. Burn injuries in critically ill patients trigger a systemic inflammatory response that leads to hypermetabolism, catabolism, and skeletal muscle mass loss, accompanied by an increase in energy expenditure and the accelerated release of muscle protein substrates, resulting in accelerated muscle mass loss [31]. This additional factor may have also contributed to the exacerbation of muscle mass loss in this group, influencing the study results.

Likewise, body composition may have influenced our findings. The relatively high BMI in our cohort suggests a high prevalence of overweight/obesity, and the potential presence of sarcopenic obesity should be considered; this phenotype has been associated with poorer physical performance and adverse outcomes in older adults [32,33]. Accordingly, variability in strength and functional status measures may reflect body composition in addition to chronological age [32]. From a methodological standpoint, increased subcutaneous adiposity may reduce the delineation of sonographic tissue planes and, if excessive transducer compression is applied, may lead to an underestimation of muscle thickness. In our study, we used standardized anatomical landmarks, a high-frequency linear transducer, and image acquisition with minimal pressure (liberal gel application and avoidance of compression), consistently with published methodological recommendations [18,34]. Nonetheless, given the pilot nature and small sample size, we cannot fully disentangle the independent effects of advanced age from those of body composition; therefore, future studies should incorporate direct adiposity assessments (e.g., waist circumference, DXA, CT, or bioimpedance) and/or adjust statistical models for adiposity markers and sarcopenic obesity.

Regarding handgrip strength, the low values obtained at awakening (~7–8 kg) for participants aged 60–75 years and those in the older 80+ group, respectively, align with findings from previous studies that observe, along with muscle mass loss, a decline in muscle strength [24,25,27,35]. Martins et al. [36] reported higher handgrip strength at ICU discharge compared with awakening for all three of their study groups—young adults, adults, and older adults—noting that strength gains were lower in the older adult group. These results are similar to those found in our study and support a less pronounced increase in the older age groups.

For the older 80+ group, the handgrip strength values at discharge (10 kg for men and 6 kg for women) indicated intensive care unit-acquired weakness (ICU-AW) (<11 kg for men and <7 kg for women) [21] (Supplementary Table S2).

These results are particularly relevant, as handgrip strength has been identified as the main determinant of sarcopenia and frailty [37].

Notably, when analyzing the values obtained at awakening, the group aged 60–75 years had a lower mean value than the older 80+ group (6.63 vs. 7.91 kg), which contradicts what has been reported in previous studies [18,36,38,39]. When examining the results individually, an outlier (20 kg) was identified, which deviates significantly from the observed data range and could influence the results.

Regarding the peripheral muscle strength values measured using the MRC-SS scale, the results obtained in our study are consistent with those reported by Silva et al. [18] and Martínez Cruz et al. [38], who demonstrated an improvement in muscle strength from awakening to ICU discharge.

Both groups evaluated in our study had an average ICU discharge score below 48 points, the established threshold for diagnosing ICU-AW [7]. Notably, the older 80+ group stood out for presenting an average score of 35.7 points at discharge, which indicates severe muscle weakness [21] (Supplementary Table S2).

As with the analysis of muscle mass, no previous studies have reported specific data on patients in the older 80+ group. In addition, the decline in muscle function has significant clinical implications, as it is associated with increased patient dependency, a higher risk of falls, and longer hospital stays. These findings highlight the importance of planning specific and individualized therapeutic interventions to address the physiological changes in this population [40].

Regarding the functional status results measured using the FSS-ICU scale, our findings are consistent with those reported by Andrade-Junior et al. [26] and Silva-Gutiérrez et al. [18], who noted that despite the progressive loss of muscle mass experienced by ICU patients, there is a trend toward improved functional status by the time of ICU discharge. These improvements in functional status, observed from awakening to ICU discharge, suggest that other neurological or physiological adaptations may mediate these functional gains [41]. Martins et al. [36] reported significant differences in FSS-ICU scores among young adults, adults, and older adults, both at awakening and at ICU discharge. While all groups showed increased scores at discharge, the gains were smaller in the older adult group, findings that align with the results observed in our study (Supplementary Table S2).

The results presented above highlight the importance of a comprehensive assessment that considers the biological, functional, psychological, and social heterogeneity of older adults, especially those in the older 80+ group [42]. This highlights the need to develop personalized therapeutic plans and requirements, where the focus extends beyond short- or medium-term mortality as the primary outcome and instead prioritizes functional recovery and quality of life as key outcome variables [43]. These results also support early, serial assessment using ultrasound, complemented by early mobilization in older adults. Moreover, the greater loss of muscle mass between ICU admission and awakening identifies a critical therapeutic window amenable to intervention. Consequently, in the older 80+ group, these findings support a more intensive, individualized rehabilitation strategy, with particular emphasis on discharge planning and post-ICU rehabilitation.

Therefore, we recommend implementing early mobilization and rehabilitation that is protocolized, progressive, and individualized, ideally within the first 48–72 h of ICU admission. This approach should be complemented by mobilization-assist devices (e.g., MotoMed), and when active mobilization is not feasible—due to sedation, limited cooperation, or clinical instability—the use of neuromuscular electrical stimulation (NMES) should be considered as an alternative to promote muscle activation and mitigate losses in muscle mass and strength [44].

In addition, we emphasize the need to strengthen multidisciplinary care through the implementation of structured bundles such as the ABCDEF bundle and/or the PADIS guidelines, which provide an operational framework to reduce deep sedation and optimize the management of pain, agitation, delirium, and immobility [45,46]. Finally, early re-initiation of nutritional support should also be prioritized as an essential component of a multimodal strategy aimed at preserving muscle mass and promoting functional status recovery [47].

This study provides original evidence in critically ill older adults in the older 80+ group by combining serial ultrasound measurements with longitudinal assessment of strength and functional status trajectories, quantifying the magnitude and temporal course of decline, and linking structural parameters to functional outcomes at ICU discharge, thereby enabling hypothesis generation regarding the morphofunctional trajectory in critically ill older adults and the quantification of the temporal dynamics of decline—dimensions that are poorly documented.

The present study has limitations. First, we included a small sample size, which may have reduced the statistical power to detect between-group differences. We emphasize that, given the exploratory nature of this study, extrapolation of the results should be made with caution. These findings are most directly applicable to critically ill older adults (those aged 60–75 years and those in the older 80+ group) managed in ICUs, requiring mechanical ventilation, and exposed to comparable levels of immobility and sedation. In other settings, the magnitude of morphofunctional changes may differ depending on the admission diagnosis (e.g., sepsis, postoperative status, trauma), illness severity (e.g., APACHE/SOFA), baseline frailty, nutritional status, and local sedation and mobilization practices. To strengthen generalizability in future research, we recommend multicenter designs with larger sample sizes, stratification by sex and diagnosis, adjustment for severity and/or frailty, and the use of multivariable models to estimate independent effects. Accordingly, these results should be interpreted as hypothesis-generating and as a clinical warning signal supporting early interventions, rather than as definitive effect estimates.

Second, due to the inherent design of observational studies, we were unable to control for potential confounding variables that may have influenced the outcomes related to strength, functional status, and muscle mass—such as medical diagnoses, nutritional intake, and physical therapy. Finally, we were unable to assess the presence of geriatric syndromes (e.g., frailty, malnutrition, sarcopenia), which may have particularly impacted the results in the older 80+ group.

## 5. Conclusions

Both older adult groups admitted to an ICU experience a decline in skeletal muscle mass, muscle strength, and functional status during their ICU stay, with a tendency for the decline to be greater in the older 80+ group. In addition, critically ill older groups recover muscle strength and functional status from awakening to ICU discharge, with a tendency for recovery to be smaller in the older 80+ group. These conclusions are based on trends observed in an exploratory study and require confirmation in larger trials.

## Figures and Tables

**Table 1:** Participants’ characteristics.

Variable	Total Participants n = 17 $N{}^{\circ},\;\bar{x}$ ± SD, %	Aged 60–75 n = 8 $N{}^{\circ},\;\bar{x}$ ± SD, %	Older 80+ Group n = 9 $N{}^{\circ},\;\bar{x}$ ± SD, %	*p*
Age (years)	76.7 ± 9.61	68.13 ± 5.79	84.44 ± 3.81	<0.001
Sex				
- Female	(9) 53%	(5) 62.5%	(4) 44.4%	
- Male	(8) 47%	(3) 37.5%	(5) 55.6%	
Weight (kg)	78.41 ± 9.60	78.75 ± 13.77	78.11 ± 4.28	0.89
Height (cm)	162.58 ± 7.78	161.87 ± 8.32	163.22 ± 7.72	0.73
BMI (kg/m^2^)	29.71 ± 3.91	30.03 ± 5.14	29.42 ± 2.70	0.75
APACHE II score	20.41 ± 3.92	20.62 ± 4.40	20.22 ± 3.70	0.84
Causes of ICU admission				
- Respiratory	(5) 29.4%	(4) 50%	(1) 11.1%	
- Digestive	(1) 5.8%		(1) 11.1%	
- Burn	(2) 11.6%		(2) 22.2%	
- Shock	(9) 52.9%	(4) 50%	(5) 55.5%	
Comorbidities				
- Hypertension	11	4	7	
- Diabetes	11	3	8	
- Dyslipidemy	8	1	7	
- Obesity	5		5	
≥2 Comorbidities	(12) 70.5%	(4) 50%	(5) 55%	
MV days	8.58 ± 4	9.62 ± 5.09	7.67 ± 2.69	0.33
Total days of stay in ICU	12.17 ± 7.22	13.75 ± 9.52	10.78 ± 4.49	0.41
Days from awakening to discharge	4.22 ± 4.15	4.55 ± 5.02	3.89 ± 3.95	0.23
Use of NMBA	1	1	0	
Transition achieved at discharge				
- Bed mobility	(8) 47%	(2) 25%	(6) 66.7%	
- Sitting at the edge of the bed	(7) 41.1%	(4) 50%	(3) 33.3%	
- Standing	(2) 11.1%	(2) 25%		

SD: standard deviation; n: number of individuals; BMI: body mass index; APACHE II: Acute Physiology and Chronic Health Evaluation II; MV: mechanical ventilation; ICU: Intensive Care Unit; NMBA: neuromuscular blocking agent.

## Data Availability

The datasets generated and/or analyzed during the current study are available from the corresponding author on reasonable request. The data are not publicly available due to privacy restrictions.

## Supplementary Materials

The following supporting information can be downloaded at https://www.mdpi.com/article/10.3390/healthcare14050585/s1. Figure S1: Flowchart of the participants. Figure S2: Timeline of the study and evaluations conducted. Figure S3: Differences in ultrasonography between participants aged 60 to 75 years and those in the older 80+ group. Table S1: Muscle mass of the participants at admission, awakening and when discharged from ICU. Table S2: Muscle strength and functional status of the participants at awakening and when discharged from ICU. Table S3: Normality testing (Shapiro–Wilk *p*-values).

## Abbreviations

ICU: Intensive Care Unit; BMI: Body Mass Index; HS: Handgrip Strength; MRC-SS: Medical Research Council Sum Score; FSS-ICU: Functional Status Score for the ICU; VI: Vastus Intermedius; RF: Rectus Femoris; TQ: Total Quadriceps; APACHE II: Acute Physiology and Chronic Health Evaluation II; Cross-Sectional Area (CSA); Extracorporeal Membrane Oxygenation (ECMO); Intensive Care Unit-Acquired Weakness (ICU-AW).
