# Supplementary material for: Skeletal Muscle Mass, Strength, and Functional Status in Critically Ill Older Adults Aged 60–75 and over 80 Years Old: An Exploratory Study
Source: Healthcare (Basel). 2026 Feb 26;14(5):585. doi: 10.3390/healthcare14050585 (PMC12984329; doi:10.3390/healthcare14050585)
Supplement: Supplementary file 1 [file healthcare-14-00585-s001.zip › healthcare-4070082-supplementary.pdf]

Supplementary Figure S1: Flowchart of the participants

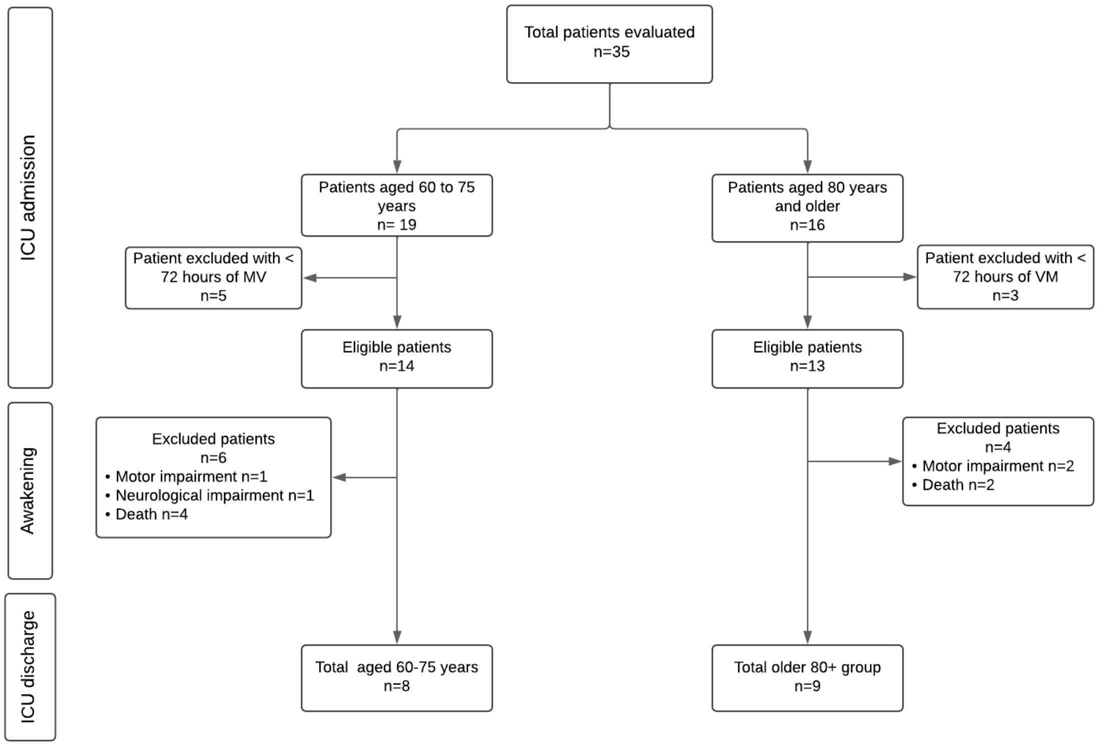

MV: mechanical ventilation.

Supplementary Figure S2: Timeline of the study and evaluations conducted

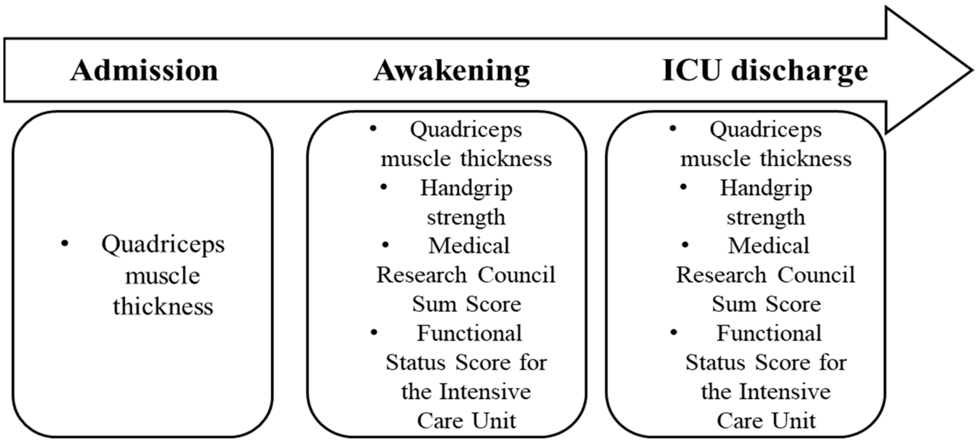

ICU: Intensive care unit.

Supplementary Figure S3: Differences in ultrasonography between participants aged 60 to 75 years and those in the older 80+ group

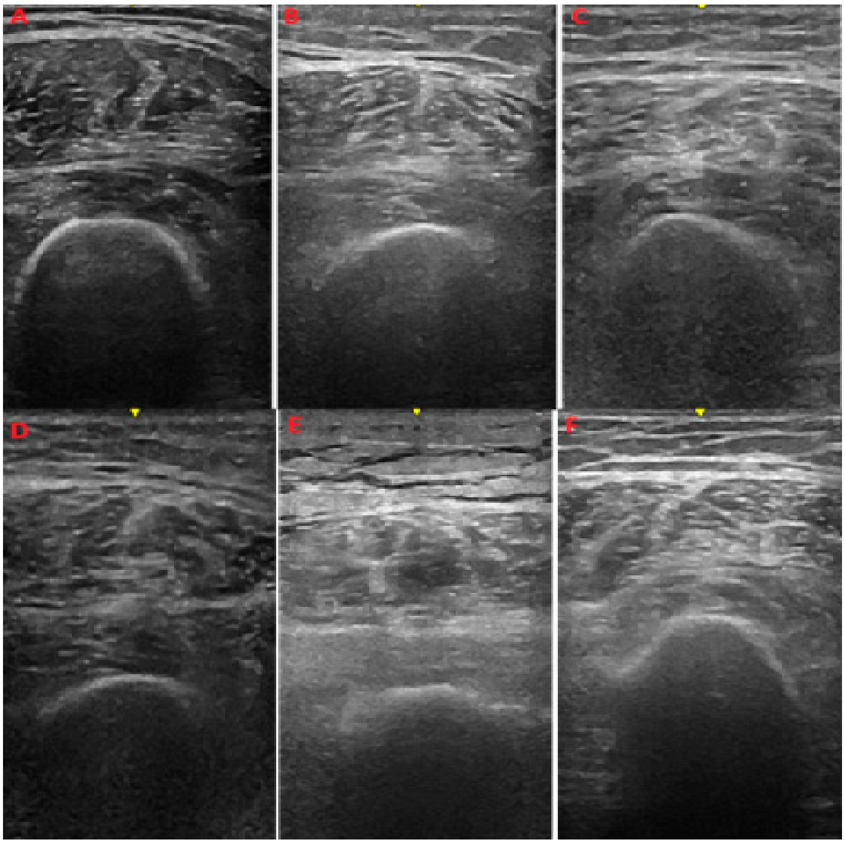

A, B, C: Ultrasound images of a participant aged 60–75 years at ICU admission, awakening, and ICU discharge, respectively, **D, E, F**: Ultrasound images of a participant in the older 80+ group at UCI admission, awakening, and ICU discharge, respectively.

Supplementary Table S1: Muscle mass of the participants at admission, awakening and when discharged from ICU.

	Group / t-test	Admission	Awakening	ICU Discharge	Delta Admission-Awakening (%)	Delta Awakening-ICU Discharge (%)	Delta Admission-ICU Discharge (%)	Time	Group	Time x group
**Quadriceps muscle thickness (cm)**	Aged 60–75	2.50 ± 0.71	2.07 ± 0.71	1.92 ± 0.71	-21.87 ± 10.14	-8.9 ± 5.45	-34.03 ± 19.34	**< 0.001**	0.09	0.30
	Older 80+ group	2.03 ± 0.17	1.71 ± 0.18	1.45 ± 0.26	-20 ± 5.95	-18.7 ± 10.32	-43.16 ± 19.91			
	t-test	0.07	0.16	0.08	0.53	0.08	0.35			
**Vastus Intermedius Muscle thickness (cm)**	Aged 60–75	0.97 ± 0.34	0.78 ± 0.30	0.73 ± 0.33	-25.42 ± 11.72	-12.80 ± 13.92	-37.11 ± 18.22	**<0.001**	0.15	0.50
	Older 80+ group	0.79 ± 0.10	0.63 ± 0.08	0.54 ± 0.08	-28.69 ± 12.40	-17.73 ± 9.75	-47.56 ± 19.83			
	t-test	0.08	0.19	0.11	0.18	0.10	0.27			
**Rectus Femoris muscle thickness (cm)**	Aged 60–75	1.41 ± 0.45	1.20 ± 0.49	1.07 ± 0.45	-23.91 ± 13.20	-11.94 ± 7.13	-37.30 ± 21.33	**<0.001**	0.13	0.56
	Older 80+ group	1.12 ± 0.16	0.99 ± 0.16	0.85 ± 0.23	-16.66 ± 8.88	-20.89 ± 14.16	-42.48 ± 33.57			
	t-test	0.08	0.23	0.21	0.40	0.15	0.71			

ICU: Intensive care unit

Supplementary Table S2: Muscle strength and functional status of the participants at awakening and when discharged from ICU

	Group / t-test	*Awakening*	*ICU Discharge*	*Delta Admission-ICU Discharge (%)*	*Time*	*Group*	*Time x group*
**Handgrip strength**	Aged 60–75	6.63 ± 3.15	8.51 ± 3.56	23.18 ± 9.02	**0.002**	0.78	0.03
	Older 80+ group	7.91 ± 5.03	8.32 ± 4.07	7.91 ± 14.50			
	t-test	0.54	0.92	**0.02**			
**Medical Research Council Sun Score**	Aged 60–75	32 ± 10.28	40.88 ± 8.52	24.35 ± 17.01	**< 0.001**	0.26	0.55
	Older 80+ group	29 ± 5.56	35.78 ± 7.32	16.00 ± 19.33			
	t-test	0.45	0.20	0.36			
**Functional Status Score for the Intensive Care Unit**	Aged 60–75	7.25 ± 3.45	12.63 ± 4.20	44.44 ± 15.82	**< 0.001**	0.58	0.03
	Older 80+ group	7.67 ± 2.55	10.56 ± 2.60	26.00 ± 23.33			
	t-test	0.77	0.23	0.08			

ICU: Intensive care unit

Supplementary Table S3: Normality testing (Shapiro–Wilk *p*-values)

	**Outcome**	**Shapiro-Wilk**		
		**1**	**2**	**3**
**Aged 60–75**	Quadriceps muscle thickness	0.82	0.24	0.08
	Handgrip strength		0.43	0.90
	Medical Research Council Sun Score		0.90	0.38
	Functional Status Score for the Intensive Care Unit		0.86	0.32
**Older 80+ group**	Quadriceps muscle thickness	0.06	0.07	0.14
	Handgrip strength		0.07	0.12
	Medical Research Council Sun Score		0.38	0.56
	Functional Status Score for the Intensive Care Unit		0.78	0.44

1: Admission; **2** Awakening; **3:** ICU discharge.
